# Who Benefits? Or, Cash, Not Criticism, for Our Hospitals

**Published:** 1910-07-23

**Authors:** 


### Editor's Letter-Box.

WHO BENEFITS? OR, CASH, NOT CRITICISM, FOR OUR HOSPITALS.

*To the Editor of* The Hospital.

A Hospital Secretary of experience writes to us :—

"When a man gives away £20 for charitable purposes it is unusual and unfortunate that he should spend nearly £10 in ostentatiously advertising his gift. When the advertisement involves a palpable puff of a weekly publication it is a matter for regret that four institutions should be 'utilised,' as they were in this instance. A reference to the files of the *Times*, the *Morning Post*, and the *Standard* will furnish the exact number of times the advertisement in question appeared. It was three, four, or five times. That the institutions did not themselves pay for the advertisement makes it all the more clear that the editor of the weekly publication was most anxious that his paper's instrumentality should not be overlooked. When The Hospital published its note 'Who Benefits?' I, for one, thought the remarks not strong enough. I also knew that at least one other journal would have severely criticised the advertisement referred to had not those who conducted it been under a personal obligation to the editor who dragged in the names of four institutions with which to strengthen his obvious puff of his own weekly publication. If the offending editor is so indignant and so certain of what the advertisement cost he will not, I presume, object to allowing the Editor of The Hospital to see the accounts, so arriving at the actual amount spent on advertising the precious £20.

"I, as a hospital secretary, should strongly object to the editor of a paper using the name of the institution I serve as many times as he liked without consulting me. It is all very well for the misguided editor to say that institutions would be 'better off with less criticism and more cash.' But, surely, institutions are *inviting* criticism when they allow a publication to use their name in advertising a sixpenny weekly. I fully expect to take up the *Times* some day and see on the front page an advertisement something like the following :—

"'The North East South West Hospital has received from a reader of the *St. Helen's Gazette* a gift of 50,000 pills made by Mr. Thomas Peecham.'"

THE HOSPITAL. July 23, 1910.

